# Genetic characterization of indigenous goat breeds in Romania and Hungary with a special focus on genetic resistance to mastitis and gastrointestinal parasitism based on 40 SNPs

**DOI:** 10.1371/journal.pone.0197051

**Published:** 2018-05-09

**Authors:** Daniela Elena Ilie, Szilvia Kusza, Maria Sauer, Dinu Gavojdian

**Affiliations:** 1 Department of Research, Research and Development Station for Sheep and Goats Caransebes, Academy for Agricultural and Forestry Sciences, Caransebes, Romania; 2 Department of Research, Research and Development Station for Bovine Arad, Academy for Agricultural and Forestry Sciences, Arad, Romania; 3 Animal Genetics Laboratory, Institute of Animal Science, Biotechnology and Nature Conservation, University of Debrecen, Debrecen, Hungary; University of Illinois, UNITED STATES

## Abstract

Goat breeding has become an important sector in Eastern Europe, with Romania and Hungary being among the major producer countries. Given the limited number of research done up-to-date concerning genetic studies of indigenous goat breeds reared in Romania and Hungary, the current preliminary study aimed to analyze the variability of genes related to mastitis and gastrointestinal parasitism by using Kompetitive Allele Specific PCR (KASP™). We studied 52 single nucleotide polymorphisms (SNPs) belonging to 19 genes in indigenous breeds from both countries, namely Banat’s White (n = 36), Carpatina (n = 35) from Romania and Hungarian Milking (n = 79) and identified 16 polymorphic SNPs among 10 genes (*PTX3*, *IL6*, *CLEC4E*, *IL8*, *IL1RN*, *IL15RA*, *TNFSF13*, *SOCS3*, *TNF* and *TLR3*) in 150 animals. Furthermore, the diversity of the studied breeds was investigated. The PIC values ranged from 0.042 to 0.691. The mean values of observed and expected heterozygosity were 0.235 and 0.246 respectively. The highest observed heterozygosity was obtained for *IL15RA* g.10343904C>T in Banat’s White (0.464), *IL15RA* g.10354813C>T in Carpatina (0.577) and *SOCS3* g.52626440T>G in Hungarian Milking (0.588). Pairwise F_ST_ values between the Romanian breeds and Romanian and Hungarian breeds were small (0.009 and 0.015), indicating the close relationship among the studied goat populations. From all the polymorphic SNPs identified, the Hungarian Milking breed showed the highest proportion of polymorphisms (100%), whereas the Carpatina breed had the lowest percentage (87.5%). The highest value of MAF was obtained for *SOCS3* g.52626440T>G (0.46), *IL15RA* g.10343904C>T (0.47), *IL15RA* g.10344025C>T (0.45), and *IL15RA* g.10354813C>T (0.42). The 16 polymorphic SNPs identified in a panel of 150 unrelated individuals belonging to three Romanian and Hungarian indigenous goat breeds could be used in future genomic based breeding schemes as markers for genetic resistance to mastitis and gastrointestinal parasitism in goat breeds found in Eastern and Central Europe.

## Introduction

The goat farming sector in Romania has been rapidly developing during the last decade. Currently, Romania holds a national flock of 1.48 million goats, according to Eurostat reports [1]. The breed structure is being dominated by the indigenous unimproved Carpatina, which represents over 90% of the goats reared in Romania. Reports concerning the breed’s performance have shown modest production levels, with milk yields estimates of 220 to 350 kg/lactation, litter size of 130–160% and growth rates in kids ranging between 90 and 110 g/day [2,3]. The second indigenous goat breed found in Romania is the Banat’s White. The breed is currently listed as endangered and included in a conservation program, with a census of 1,002 purebred does reared in 5 farms. The Banat’s White has a milk production of 350–400 kg/lactation and is highly prolific, with an average litter size of 200–225% [4].

Nowadays the national Hungarian goat population is of 81,000 heads [1] and the main production is dairy [5]. Goats are being reared under extensive low-input production systems in over 7,000 farming units, with an average flock size of roughly 15 breeding does/farm [6]. The most frequent goat breed is the Hungarian Milking [7], with the adult body weight of 40 to 60 kg in does and 60 to 90 kg in adult bucks. Reports concerning the breed’s performance have shown modest production levels, with milk yields estimates of 200 to 300 kg/lactation and the litter size of 130–150% [8,9].

In the recent decades, several studies have attempted to investigate and identify genetic variants responsible for mastitis and gastrointestinal parasitism resistance in livestock. So far, studies on the genetic structure of goat breeds and genetic basis of immune cell involved in response to intramammary pathogens were not performed in Romanian and Hungarian indigenous goat breeds. Despite the management practices, mastitis and parasitic infections are among the main health constraints for the small ruminants sector worldwide, being responsible for causing heavy production losses and poor animal welfare [10–12]. Clinical mastitis is less frequent in dairy sheep and goats (5%) compared with dairy cattle, however, subclinical mastitis has a prevalence of up to 55% [13,14]. On the other hand, gastrointestinal nematode infections are the most prevalent parasitic diseases affecting sheep and goats productivity worldwide, especially under pasture-based production conditions. The condition can generate the reduction in skeletal growth, live-weight gain and in milk yield, causing significant economic losses, reduced animal performance and even leading to mortality in severe infestations [15,16].

Knowledge on the genetic structure of goat breeds, as well as new information about the genetic basis of immune cell involved in response to pathogens, will be beneficial to understand the role of genetic variants in resistance to mastitis and gastrointestinal parasitism. Furthermore, genetic improvement schemes and conservation plans are becoming more and more important in each country for all farm species, and are being designed based on both phenotypic and genomic data. For conservation plans, there is a need for detailed knowledge of the genetic make-up of the goat breeds in Central and Eastern Europe. The aim of the current pilot study was to analyze variability of 52 SNPs found on 19 genes related to mastitis and gastrointestinal parasitism in the indigenous goat breeds from Romania and Hungary through the use of a novel and fast method, called Kompetitive Allele Specific PCR.

## Material and methods

### Ethics approval

The research activities were performed in accordance with the European Union’s Directive for animal experimentation (Directive 2010/63/UE). The experimental design, sampling collection protocols and procedures were approved by the Institutional Ethics Committee of the Research and Development Station for Sheep and Goats Caransebes (Decision no. 39 from 05 November 2015).

### Animals and DNA isolation

A total of 150 goats belonging to three Romanian and Hungarian indigenous breeds were included in the study. Hair follicles were sampled from 71 Romanian goats (Banat’s White n = 36 and Carpatina n = 35) and blood samples from 79 Hungarian goats (Hungarian Milking n = 79) were collected. The animals were selected following the criteria to be unrelated individuals and sampled from different farms (2–5 farms/breed unit) in order to reduce the genetic relationship among animals and to increase the breed representativeness. The collection sites and geographic coordinates of the studied breeds used in the study were given in S1 Table. Collection of samples was performed by authorized veterinarians and all samples were kept on 4°C until the further laboratory process. Genomic DNA was extracted following FAO/IAEA protocol [17] for the hair follicles and Zsolnai and Orbán protocol [18] for blood. DNA concentration was evaluated spectrophotometrically, with NanoDrop-2000 (Thermo Fisher Scientific Inc., MA, USA), and visually by standard agarose gel electrophoresis (1% agarose (w/v) in TBE). After extraction, all DNA samples were diluted for 50 ng and stored at -20°C until the further analysis.

### Selection of SNPs

A total of 52 SNPs belonging to 19 genes (S2 Table) have been selected to be used in Kompetitive Allele Specific PCR assay based on studies [11,19–22] involving markers for genetic resistance to gastrointestinal infection and genes associated with resistance to mammary gland infection. SNPs data were collected from the caprine Single Nucleotide Database (dbSNP) of the National Center for Biotechnology Information (NCBI).

### DNA genotyping and data analysis

Kompetitive Allele Specific PCR (KASP™, LGC Genomics, Teddington, Middlesex, UK) genotyping was performed for the bi-allelic discrimination of the selected SNPs (S2 Table). The data generated was viewed using SNP viewer software (version 1.99, Hoddesdon, UK). The raw allele calls received from LGC Genomics were analyzed with KlusterCaller software from LGC Genomics. Linkage analysis was performed by GENEPOP [23]. POPGENE 1.32 [24] was used to calculate the deviation from Hardy-Weinberg equilibrium (HWE), observed (Ho) and expected (He) heterozygosity values for each studied breed, Polymorphic Information Content (PIC) and pairwise FST values.

## Results

In this study, 52 SNPs across 19 genes were studied using the KASP genotyping assays. Details of SNPs including SNP ID, gene name, chromosome location, genomic location, functional domain of the gene and alleles at each locus are presented in S2 Table. Out of the 52 SNPs, 16 (30.77%) were found to be polymorphic, 24 (46.15%) monomorphic and 12 (23.08%) failed in our studied goat breeds (S3 Table). However, a total of 40 SNPs (monomorphic and polymorphic) were used from a total of 6,000 genotypes assayed. A total of 24 alleles and 42 genotypes were found at 16 polymorphic SNPs in the 150 studied goats. The polymorphic SNPs were found in the following 10 genes: *PTX3*, *IL6*, *CLEC4E*, *IL8*, *IL1RN*, *IL15RA*, *TNFSF13*, *SOCS3*, *TNF* and *TLR3*. From polymorphic SNPs identified, four were located in chromosome 13, two were located in each of chromosomes 5, 6, 11 and 19 and one in each of chromosomes 1, 4, 23, and 27 respectively. The monomorphic SNPs were excluded from further analysis.

For the polymorphic SNPs, the assays produced 2,142 identified allele calls and 258 unidentified allele calls with an allele call rate of 89.25% and a mean of unidentified allele calls of 10.75%. All polymorphic SNPs revealed more than 84% identified allele calls/SNP. Among the polymorphic SNPs, twelve SNPs (75%,) were located within the coding region of the gene (*CLEC4E* g.93527308C>T, *IL8* g.86041868A>G, *IL8* g.86040123G>A, *IL1RN* g.46358256A>G, *IL1RN* g.46353777G>T, *IL15RA* g.10343904C>T, *IL15RA* g.10344025C>T, *IL15RA* g.10354726G>A, *IL15RA* g.10354813C>T, *TNFSF13* g.26523480A>G, *TNF* g.26141981T>A and *TLR3* g.14987931C>G), two were located in introns (*PTX3* g.108076746C>T and *SOCS3* g.52626440T>G), one in the 3′UTRs (*IL6* g.29257937T>C) and one was an upstream variant (*CLEC4E* g.93538087T>C). However, when the experiment was designed, non-synonymous SNPs were mainly chosen in order to increase the probability that there would be a change in the characteristic of the proteins encoded for the investigated genes. Furthermore, for polymorphic SNPs located on the same chromosome (BTA13) and the same gene (*IL15RA*) a linkage analysis was performed and shown that all four SNPs are independent.

The genetic indices of Ho, He, PIC and FST values for different breeds under study were calculated and shown in Table 1. PIC values also revealed that rs635583012 / *SOCS3* g.52626440T>G (0.640–0.693), rs661943224 / *IL15RA* g.10343904C>T (0.659–0.693), rs648293427 / *IL15RA* g.10344025C>T (0.632–0.693), *IL15RA* g.10354813C>T (0.640–0.691), rs661914424 / *TLR3* g.14987931C>G (0.583–0.664) and *IL1RN* g.46353777G>T (0.467–0.417) are the most polymorphic markers.

**Table 1:** Main diversity indices (polymorphic information content (PIC), expected (He) and observed (Ho) heterozygosity) for the polymorphic SNPs.

					Banat's White			Carpatina			Romanian goats				Hungarian Milking			Altogether			
SNP	Locus	Chromosome	Allele 1/2	Aminoacid change	PIC	He	Ho	PIC	He	Ho	PIC	FST	He	Ho	PIC	He	Ho	PIC	FST	He	Ho
rs661165283	*TNF* g.26141981T>A	23	A/T	G215L	0.377	0.222	0.250	0.393	0.235	0.133	0.385	0.000	0.227	0.194	0.404	0.241	0.253	0.395	0.000	0.234	0.227
rs661914424	*TLR3* g.14987931C>G	27	C/G	A445G	0.664	0.479	0.414	0.583	0.401	0.539	0.632	0.014	0.444	0.473	0.659	0.469	0.411	0.648	0.011	0.458	0.438
rs635583012	*SOCS3* g.52626440T>G	19	G/T		0.640	0.455	0.355	0.693	0.509	0.444	0.675	0.021	0.486	0.397	0.693	0.503	0.588	0.691	0.024	0.499	0.500
rs646307174	*IL6* g.29257937T>C	4	C/T		0.353	0.204	0.226	0.158	0.073	0.074	0.273	0.021	0.144	0.155	0.119	0.050	0.051	0.192	0.027	0.091	0.096
rs669680484	*PTX3* g.108076746C>T	1	C/T		0.000	0.000	0.000	0.000	0.000	0.000	0.000	0.000	0.000	0.000	0.068	0.025	0.025	0.042	0.009	0.014	0.014
rs655338449	*CLEC4E* g.93527308C>T	5	C/T	T175A	0.280	0.151	0.161	0.173	0.082	0.083	0.237	0.007	0.120	0.127	0.309	0.170	0.186	0.279	0.007	0.148	0.160
rs659842900	*IL1RN* g.46358256A>G	11	A/G	I61V	0.353	0.204	0.097	0.317	0.177	0.192	0.337	0.001	0.190	0.140	0.305	0.166	0.156	0.319	0.001	0.176	0.149
rs640582069	*IL1RN* g.46353777G>T	11	G/T	M1R	0.517	0.339	0.303	0.517	0.339	0.364	0.517	0.000	0.337	0.333	0.467	0.294	0.329	0.491	0.002	0.313	0.331
rs667413402	*IL8* g.86041868A>G	6	A/G	K83R	0.189	0.091	0.094	0.000	0.000	0.000	0.117	0.024	0.049	0.050	0.038	0.013	0.013	0.075	0.025	0.029	0.029
rs665173888	*IL8* g.86040123G>A	6	A/G	A18T	0.301	0.166	0.179	0.483	0.311	0.292	0.395	0.020	0.235	0.231	0.389	0.230	0.211	0.392	0.014	0.231	0.219
rs661943224	*IL15RA* g.10343904C>T	13	C/T	R340H	0.693	0.508	0.464	0.659	0.475	0.519	0.683	0.013	0.494	0.491	0.666	0.477	0.392	0.691	0.040	0.500	0.434
rs648293427	*IL15RA* g.10344025C>T	13	C/T	G300S	0.676	0.492	0.444	0.632	0.449	0.500	0.658	0.007	0.470	0.472	0.693	0.503	0.377	0.687	0.021	0.496	0.415
rs647408958	*IL15RA* g.10354726G>A	13	A/G	H249Y	0.079	0.030	0.030	0.079	0.030	0.030	0.079	0.000	0.030	0.030	0.038	0.013	0.013	0.058	0.001	0.021	0.021
rs635969404	*IL15RA* g.10354813C>T	13	C/T	A220T	0.640	0.455	0.419	0.657	0.473	0.577	0.648	0.001	0.460	0.491	0.691	0.501	0.480	0.679	0.013	0.488	0.485
rs669561078	*TNFSF13* g.26523480A>G	19	A/G	H116R	0.081	0.031	0.031	0.095	0.039	0.039	0.087	0.000	0.034	0.035	0.223	0.111	0.117	0.170	0.013	0.079	0.082
rs669986850	*CLEC4E* g.93538087T>C	5	C/T		0.143	0.064	0.065	0.279	0.150	0.160	0.209	0.011	0.102	0.107	0.334	0.187	0.208	0.285	0.013	0.152	0.165
**Means**					**0.374**	**0.243**	**0.221**	**0.357**	**0.234**	**0.247**	**0.371**	**0.009**	**0.239**	**0.233**	**0.381**	**0.247**	**0.238**	**0.381**	**0.014**	**0.246**	**0.235**

Expected and observed heterozygosity values detected in two Romanian and one Hungarian breeds were similarly low (<0.600). The mean global observed and expected heterozygosity was 0.235 and 0.246 respectively. The highest observed heterozygosity was obtained for rs661943224 / *IL15RA* g.10343904C>T in Banat’s White (0.464), rs635969404 / *IL15RA* g.10354813C>T in Carpatina (0.577) and rs635583012 / *SOCS3* g.52626440T>G in Hungarian Milking (0.588) indicating high levels of within-population diversity.

Pairwise FST values between the Romanian breeds and Romanian and Hungarian breeds were small ranging within the range of 0.0–0.024 and 0.0–0.040 respectively, indicating the close relationship among the studied breeds (Table 1). Thereby, low genetic differentiation between breeds (FST) were obtained, of 0.9% and 1.5%, respectively. With the Romanian breeds showing less differentiation. Moderate genetic differentiation was observed only for one SNP (rs661943224 / *IL15RA* g.10343904C>T) in Romanian breeds/Hungarian Milking (0.040).

The Hardy-Weinberg equilibrium and genotype and allele frequencies of the 16 SNPs were also studied in each of the investigated goat breeds and are shown in Table 2. All breeds were found to be in equilibrium (*P*>0.05) except one SNP in each breed (rs659842900 / *IL1RN* g.46358256A>G in Banat’s White, rs661165283 / *TNF* g.26141981T>A in Carpatina and rs648293427 / *IL15RA* g.10344025C>T in Hungarian Milking).

**Table 2:** Allele, genotype frequencies and HWE test for the polymorphic SNPs.

SNP ID/ Locus	Breed	Genotype frequency			Allele frequency		HWE
		**AA**	**AT**	**TT**	**A**	**T**	
rs661165283	Banat's White	0.000	0.250	0.750	0.13	0.87	0.453
*TNF* g.26141981T>A	*Carpatina*	*0*.*067*	*0*.*133*	*0*.*800*	*0*.*13*	*0*.*87*	*0*.*012*
	Romanian breeds	0.032	0.194	0.774	0.13	0.87	0.237
	Hungarian Milking	0.013	0.253	0.734	0.14	0.86	0.654
	Altogether	0.021	0.227	0.752	0.13	0.87	0.717
		**CC**	**CG**	**GG**	**C**	**G**	
rs661914424	Banat's White	0.172	0.414	0.414	0.38	0.62	0.454
*TLR3* g.14987931C>G	Carpatina	0.000	0.538	0.462	0.27	0.73	0.072
	Romanian breeds	0.091	0.473	0.436	0.33	0.67	0.632
	Hungarian Milking	0.164	0.411	0.425	0.37	0.63	0.284
	Altogether	0.133	0.438	0.430	0.35	0.65	0.616
		**GG**	**GT**	**TT**	**G**	**T**	
rs635583012	Banat's White	0.484	0.355	0.161	0.66	0.34	0.210
*SOCS3* g.52626440T>G	Carpatina	0.296	0.444	0.259	0.52	0.48	0.503
	Romanian breeds	0.397	0.397	0.207	0.59	0.41	0.156
	Hungarian Milking	0.191	0.588	0.221	0.49	0.51	0.161
	Altogether	0.286	0.500	0.214	0.54	0.46	0.990
		**CC**	**CT**	**TT**	**C**	**T**	
rs646307174	Banat's White	0.000	0.226	0.774	0.11	0.89	0.515
*IL6* g.29257937T>C	Carpatina	0.000	0.074	0.926	0.04	0.96	0.889
	Romanian breeds	0.000	0.155	0.845	0.08	0.92	0.547
	Hungarian Milking	0.000	0.051	0.949	0.03	0.97	0.841
	Altogether	0.000	0.096	0.904	0.05	0.95	0.575
		**CC**	**CT**	**TT**	**C**	**T**	
rs669680484	Banat's White	1 000	0.000	0.000	1.000		monomorphic
*PTX3* g.108076746C>T	Carpatina	1 000	0.000	0.000	1.000		monomorphic
	Romanian breeds	1 000	0.000	0.000	1.000		monomorphic
	Hungarian Milking	0.975	0.025	0.000	0.99	0.01	0.936
	Altogether	0.986	0.014	0.000	0.99	0.01	0.952
		**CC**	**CT**	**TT**	**C**	**T**	
rs655338449	Banat's White	0.839	0.161	0.000	0.92	0.08	0.665
*CLEC4E* g.93527308C>T	Carpatina	0.917	0.083	0.000	0.96	0.04	0.881
	Romanian breeds	0.873	0.127	0.000	0.94	0.06	0.642
	Hungarian Milking	0.814	0.186	0.000	0.91	0.09	0.412
	Altogether	0.840	0.160	0.000	0.92	0.08	0.344
		**AA**	**AG**	**GG**	**A**	**G**	
rs659842900	*Banat's White*	*0*.*839*	*0*,*097*	*0*.*065*	*0*.*89*	*0*.*11*	*0*.*002*
*IL1RN* g.46358256A>G	Carpatina	0.808	0.192	0.000	0.90	0.10	0.631
	*Romanian breeds*	*0*.*825*	*0*.*140*	*0*.*035*	*0*.*89*	*0*.*11*	*0*.*040*
	Hungarian Milking	0.831	0.156	0.013	0.91	0.09	0.567
	Altogether	0.828	0.149	0.022	0.90	0.10	0.075
		**GG**	**TG**	**TT**	**G**	**T**	
rs640582069	Banat's White	0.636	0.303	0.061	0.79	0.21	0.525
*IL1RN* g.46353777G>T	Carpatina	0.606	0.364	0.030	0.79	0.21	0.672
	Romanian breeds	0.621	0.333	0.045	0.79	0.21	0.932
	Hungarian Milking	0.658	0.329	0.013	0.82	0.17	0.273
	Altogether	0.641	0.331	0.028	0.81	0.19	0.477
		**AA**	**AG**	**GG**	**A**	**G**	
rs667413402	Banat's White	0.906	0.094	0.000	0.95	0.05	0.822
*IL8* g.86041868A>G	Carpatina	1 000	0.000	0.000	1.000		monomorphic
	Romanian breeds	0.950	0.050	0.000	0.98	0.02	0.872
	Hungarian Milking	0.987	0.013	0.000	0.99	0.01	1.000
	Altogether	0.971	0.029	0.000	0.99	0.01	0.882
		**AA**	**AG**	**GG**	**A**	**G**	
rs665173888	Banat's White	0.000	0.179	0.821	0.09	0.91	0.645
*IL8* g.86040123G>A	Carpatina	0.042	0.292	0.667	0.19	0.81	0.747
	Romanian breeds	0.019	0.231	0.750	0.13	0.87	0.887
	Hungarian Milking	0.026	0.211	0.763	0.13	0.87	0.449
	Altogether	0.023	0.219	0.758	0.13	0.87	0.535
		**CC**	**CT**	**TT**	**C**	**T**	
rs661943224	Banat's White	0.286	0.464	0.250	0.52	0.48	0.640
*IL15RA* g.10343904C>T	Carpatina	0.370	0.519	0.111	0.63	0.37	0.628
	Romanian breeds	0.327	0.491	0.182	0.57	0.43	0.964
	Hungarian Milking	0.189	0.392	0.419	0.39	0.61	0.123
	Altogether	0.248	0.434	0.318	0.47	0.53	0.135
		**CC**	**CT**	**TT**	**C**	**T**	
rs648293427	Banat's White	0.370	0.444	0.185	0.59	0.41	0.609
*IL15RA* g.10344025C>T	Carpatina	0.423	0.500	0.077	0.67	0.33	0.550
	Romanian breeds	0.396	0.472	0.132	0.63	0.37	0.973
	*Hungarian Milking*	*0*.*312*	*0*.*377*	*0*.*312*	*0*.*50*	*0*.*50*	*0*.*026*
	Altogether	0.346	0.425	0.238	0.55	0.45	0.063
		**AA**	**AG**	**GG**	**A**	**G**	
rs647408958	Banat's White	0.000	0.030	0.970	0.02	0.98	1.000
*IL15RA* g.10354726G>A	Carpatina	0.000	0.030	0.970	0.02	0.98	1.000
	Romanian breeds	0.000	0.300	0.970	0.02	0.98	0.930
	Hungarian Milking	0.000	0.013	0.987	0.01	0.99	1.000
	Altogether	0.000	0.021	0.979	0.01	0.99	0.918
		**CC**	**CT**	**TT**	**C**	**T**	
rs635969404	Banat's White	0.129	0.419	0.452	0.34	0.66	0.654
*IL15RA* g.10354813C>T	Carpatina	0.077	0.577	0.346	0.37	0.63	0.251
	Romanian breeds	0.105	0.491	0.404	0.35	0.65	0.599
	Hungarian Milking	0.227	0.480	0.293	0.47	0.53	0.713
	Altogether	0.174	0.485	0.341	0.42	0.58	0.941
		**AA**	**AG**	**GG**	**A**	**G**	
rs669561078	Banat's White	0.969	0.031	0.000	0.98	0.02	1.000
*TNFSF13* g.26523480A>G	Carpatina	0.962	0.038	0.000	0.98	0.02	1.000
	Romanian breeds	0.966	0.034	0.000	0.98	0.02	0.925
	Hungarian Milking	0.883	0.117	0.000	0.94	0.06	0.609
	Altogether	0.919	0.081	0.000	0.96	0.04	0.639
		**CC**	**CT**	**TT**	**C**	**T**	
rs669986850	Banat's White	0.000	0.065	0.935	0.03	0.97	0.896
*CLEC4E* g.93538087T>C	Carpatina	0.000	0.160	0.840	0.08	0.92	0.709
	Romanian breeds	0.000	0.107	0.893	0.05	0.95	0.700
	Hungarian Milking	0.000	0.208	0.792	0.10	0.90	0.326
	Altogether	0.000	0.165	0.835	0.08	0.92	0.311

*Italics*: No HWE; P<0.05

Levels of polymorphism were generally low in all studied breeds. The homozygous genotypes were more frequent than heterozygous in most cases. On average, from polymorphic SNPs identified, the Hungarian Milking breed showed the highest proportion of polymorphic SNPs (100%), whereas the Carpatina breed had the lowest proportion of polymorphic SNPs (87.5%) and presented fixed alleles in a number of 2 SNPs (rs669680484 and rs667413402). From the 16 polymorphic SNPs, four (25%) were found with an overall frequency of the rare allele lower than 5% (rs669680484/*PTX3*, rs667413402/*IL8*, rs647408958/ *IL15RA* and rs669561078/ *TNFSF13*). The frequencies of major alleles ranged from 0.53 for rs661943224/*IL15RA* to 0.99 (SNPs with rare allele lower than 5%). The highest value of minor allele frequency (MAF) was obtained for *TLR3* g.14987931C>G (0.35), *SOCS3* g.52626440T>G (0.46), *IL15RA* g.10343904C>T (0.47), *IL15RA* g.10344025C>T (0.45), and *IL15RA* g.10354813C>T (0.42).

## Discussion

Kompetitive Allele Specific PCR technology was used to convert a set of 52 SNPs into assays for genetic characterization of indigenous goat breeds reared in Romania and Hungary with a special focus on genetic resistance to the mammary gland and gastrointestinal infections. Among the 52 SNPs, a number of 12 (23.08%) failing development and 40 (76.92%) were successfully genotyped of which 24 (46.15%) were monomorphic across Banat’s White, Carpatina and Hungarian Milking goat breeds. Current findings are in accordance with those previously reported in studies where the KASP assay success rates were ranging from 78.5% [25] to 80.9% [26]. With the failed and monomorphic assays removed, 16 SNPs (30.77%) were further validated and used for the current study. Of the 16 SNPs, a percent of 75% (12 SNPs) met the criteria for analysis (MAF ≥ 5% and call rate > 89%).

### Genetic resistance to the mammary gland and gastrointestinal infection

The present study found 16 polymorphic SNPs across 10 genes that are relevant in pathways associated with parasite and mammary gland infection or are involved in production traits or have a potential contribution to an important metabolic condition. However, functional genes, such as *CLEC4E*, *IL6*, *IL8*, *IL1RN*, *IL15RA*, *PTX3*, *SOCS3*, *TNF*, *TNFSF13* and *TLR3* are related to host resistance to disease in different farm species.

The *CLEC4E* is expressed on the surface of macrophages [27] and play an important role in recognition of bacterial glycolipids by the immune system being an immune response gene associated with genetic resistance and susceptibility to a wide array of diseases [21]. Bhuiyan *et al*. [22] showed that *CLEC4E* was a down-regulated gene involved in immune response system in susceptible goats to nematodes infection. In others studies on goats, *CLEC4E* was up-regulated when was investigated in the *in vivo* transcriptional response of mammary epithelial cells at the early stages of infection with *Staphylococcus aureus* [21]. In this paper, were analyzed four SNPs in *CLEC4E* gene and found two to be polymorphic (*CLEC4E* g.93527308C>T and *CLEC4E* g.93538087T>C) in all three studied goat breed.

Pentraxin 3 (*PTX3*) is a glycoprotein expressed by diverse cell types upon primary inflammatory stimuli such as those mediated by *IL-1β*, *TNFα* and agonists of *TLRs* [28,29]. The *PTX3* gene has as a primary function the regulation of innate resistance to pathogens and inflammatory reactions and acts as antimicrobial agents that could assist defense of the mammary gland against chronic and subclinical infections [11]. In our study, five SNPs were investigated in intron regions of *PTX3* gene, of which four presented fixed alleles and one was found polymorphic (*PTX3* g.108076746C>T). Moreover, the results showed that polymorphic SNP at the *PTX3* gene was not presented in Carpatina or Banat’s White goat breeds. Different studies showed that the *PTX3* represents the first line of immune defense in udder being significantly up-regulated in response to *Staphylococcus aureus* infection in goats [11,30]. Other studies conducted in different farm species revealed that the phenotype of *PTX3* gene plays a protective role against several types of harmful microorganisms [29].

*SOCS3* is a member of the suppressors of cytokine signaling (*SOCS*) family of proteins that have a negative effect on cytokine signaling [31]. Brenaut *et al*. [21] studied the contribution of mammary epithelial cells to the immune response during early stages of a bacterial infection to *Staphylococcus aureus* in goats and revealed that *SOCS3* is a highly regulated gene with a high degree of outward connectivity to other genes that are for the most up-regulated. Moreover, polymorphism of *SOCS3* gene was associated with mammary development pathway [32] and somatic cell score trait in cattle [33,34]. In the current study, the *SOCS3* g.52626440T>G was polymorphic in all three studied breeds, with the highest frequency of T allele (0.51) being found in the Hungarian Milking goats.

The level of some cytokines such as *TNF-α*, *IL6* and *IL8* are reported to increase during infections [35]. The interleukin-8 (*IL8* or chemokine (C-X-C motif) ligand 8, *CXCL8*) is a chemokine produced by several cell types such as macrophages epithelial and endothelial cells [36]. This chemokine is one of the major mediators of the inflammatory response and acts on CXCR1 and CXCR2 receptors [37]. In humans, the gene polymorphism studies indicate that regions within the gene, others than promoter region, may contribute to CXCL8 production and, potentially, susceptibility to certain infectious diseases [38]. In cattle, analyzing of key molecules of the innate immune system in mammary epithelial cells has been revealed that the chemokine IL8 showed a significant increase in expression level in *Escherichia coli* as well as in *Staphylococcus aureus* -affected cells [39]. Brenaut *et al*. [21] revealed that mammary epithelial cells play an important role in the recruitment and activation of inflammatory cells through the *IL*8 signaling pathway. Furthermore, several researches suggest that different polymorphisms of the *IL8* gene are associated with increased risk of infection from *Escherichia coli* and *Helicobacter pylori* and due to infection, have elevated inflammatory responses and/or more clinically significant disease [40,41]. In the present research, both investigated SNPs in *IL8* were polymorphic (*IL8* g.86041868A>G and *IL8* g.86040123G>A).

The interleukin-6 (*IL6*) is a cytokine with a wide range of biological activities that play an important role in immune regulation and inflammation. Data from several studies on humans suggest that *IL6* plays a critical role in the B cell hyperactivity and immunopathology of several diseases [42,43]. In our study, *IL6 g*.*29257937T>C* was polymorphic in all three studied breeds, with the highest value of MAF found in Banat’s White goats (0.11).

The tumor necrosis factor (TNF), known as TNFα or TNF alpha, is involved in systemic inflammation. The primary role of TNF is in the regulation of immune cells. A recent study examined the diversity in the *TNF-*α exon 4 and 3'UTR in native Chinese domestic goats and three SNPs in the 3'UTR region [44] were identified. However, an investigation on North American goat breeds revealed that the same analyzed SNPs of *TNF-*α were monomorphic [45]. In cattle, the role of TNF-α has been reported also in an acute mastitis [46]. Here, the locus TNF g.26141981T>A was polymorphic in all breeds, having the same value of MAF (0.13).

The interleukin-1 receptor antagonist (IL-1RA), encoded by the *IL1RN*, belongs to the interleukin 1 cytokine family [47]. The protein encoded by this gene modulates a variety of interleukin 1 related to the immune and inflammatory responses [48]. In humans, the polymorphisms of this gene were reported to be associated with different diseases [49]. Brenaut *et al*. [21] investigated in goats the *in vivo* transcriptional response of mammary epithelial cells at the early stages of infection with *Staphylococcus aureus* and found a highly increased level of *IL1RN* expression. In our research, were analyze four non-synonymous SNPs in *IL1RN* gene and found *IL1RN* g.46358256A>G and *IL1RN* g.46353777G>T to be polymorphic in all studied animals.

The *IL15RA* gene encodes a cytokine receptor that specifically binds interleukin 15 (IL15) with high affinity. Among related pathways are the IL-15 signaling pathways and its primary biological effects on different immune cell types and innate immune systems. In humans, the SNPs in *IL15RA* were correlated with macro-pathogen richness and might indicate selection for improved intestinal clearance of nematodes [50]. However, no studies on goats were published up-to-date related to polymorphism in *IL15RA*. Current findings revealed four polymorphic SNPs in *IL15RA* gene (*IL15RA* g.10343904C>T, *IL15RA* g.10344025C>T, *IL15RA* g.10354726G>A and *IL15RA* g.10354813C>T) out of ten analyzed non-synonymous SNPs in exon regions. The values of MAF in three of *IL15RA* loci was >42%, those markers are therefore high informative.

The SNPs investigated in the present study, such as *CLEC4E*, *IL6*, *IL8*, *IL1RN*, *IL15RA*, *PTX3*, *SOCS3*, *TNF*, *TNFSF13* and *TLR3*, may be potential markers for genetic resistance to the mammary gland and gastrointestinal parasite infection in goats. Several studies revealed a number of polymorphisms of these genes that proved to have crucial roles in pathogen recognition and influencing additional immunological processes and therefore playing an important role in infections. However, to date, the full importance of these SNPs variations is unclear and therefore, an increased understanding of those genes variation in each goat breed is important for determining the genes associated with parasite and mammary gland infection.

This study provides the first view of the polymorphisms in genes related to mastitis and parasite infection in Romanian and Hungarian goat breeds. By KASP assay was possible to identify 16 polymorphic SNPs in ten genes related to mastitis and parasite infection in Banat’s White, Carpatina and Hungarian Milking goat breeds. Although the use of this SNPs needs further studies related to marker associations and their marker-quantitative trait locus phase relationships in each population, in order to specify each SNP effect, the results obtained could prove valuable and contribute to future molecular markers studies related to parasites and mammary gland infections in goats.

### Genetic diversity among Romanian and Hungarian indigenous goat breeds

The results of the current study showed a low genetic differentiation (F_ST_) among studied breeds. The mean F_ST_ ranging from 0.009 for Banat’s White/Carpatina to 0.014 for Romanian breeds/Hungarian Milking. Those values of genetic differentiation among goat breeds are lower than values reported in previous studies on eight goat breeds from different European regions genotyped for 27 SNPs, where the F_ST_ values were variable within the range of 0.004–0.224 [19] or for 16 breeds from Italy, Albania and Greece assessed by 27 SNPs that revealed an overall F_ST_ of 0.063 [51]. Results obtained in the present research are comparable with other studies where the lower F_ST_ values were found among Argentinean goat populations [52]. However, the low level of genetic differentiation in the studied goat breeds could be the result of common origin, given the common border that Hungary and Romania have, and the shared common history.

For the SNPs markers, the PIC values in Romanian and Hungarian indigenous goat breeds ranged from 0.000 to 0.693 with an average value of 0.381. When individual PIC values were examined it was observed that a substantial portion (62,5%) of SNPs provided a moderate level of information (PIC≤0.50). Four SNPs (*PTX3* g.108076746C>T, *IL8* g.86041868A>G, *IL15RA* g.10354726G>A and *TNFSF13* g.26523480A>G) possess low genetic diversity in all studied goat breeds, indicating that these loci are not suggested to be effective in evaluating genetic resources of the studied goat breeds. Low PIC values were earlier reported in Korean goats [53] using microsatellite analysis. However, due to bi-allelic nature of SNPs, their PIC values can result lower. Approximately 37% of the SNPs used in the present study were informative (PIC≥0.50). Our results revealed that *SOCS3* g.52626440T>G, *IL15RA* g.10343904C>T, *IL15RA* g.10344025C>T and *TLR3* g.14987931C>G are the most polymorphic markers and therefore can be utilized for molecular characterization of the studied goat breeds.

To conclude, KASP technologies were used in current research in order to investigate a total of 52 SNPs belonging to 19 genes involved in genetic resistance to the mammary gland and gastrointestinal infection. Almost the polymorphic SNPs investigated were non-synonymous SNPs that suggested their functional role in the immune response and inflammation. The results obtained in the present study may be a further step with respect to the development of SNP genotyping assay for genetic resistance to the mammary gland and gastrointestinal infection.

## Supporting information

S1 Table The collection sites and geographic coordinates of the Romanian and Hungarian goat breeds included in the study. (DOC)

S2 Table Details of genes, chromosome location and genomic location at 52 SNP loci under study. (DOCX)

S3 Table Success ratio of 52 SNPs investigated through KASP assay for 150 samples from Banat’s White, Carpatina and Hungarian Milking goat breeds. (DOCX)

## References

[1] Eurostat. Goats population–annual data [Internet] 2017 [cited 23 Aug 2017]. Available from: http://ec.europa.eu/eurostat/data/database

[2] Voia S, Padeanu I, Daraban S, Mot T, Dronca D, Pet I, et al Study regarding goat milk composition and the growth rate in kids of Carpatina Goat Breed. Scientific Pap Anim Sci Biotechnol. 2010; 43(2):324–327

[3] Pascal C, Padeanu I, Calin I, Daraban S, Nacu G, 2011, Researches related to meat yield aptitudes of Carpatina breed reared in Romania, Scientific Pap Anim Sci Series, 55:328–331

[4] Sauer M, Padeanu I, Dragomir N, Ilisiu E, Rahmann G, Sauer WI, et al Organic goat meat production in less favoured areas of Romania. Landbauforsch Appl Agric Forestry Res. 2015; 1(65): 59–64.

[5] Németh T, Kukovics S, Baranyai G. A Magyarországon tartott kecskefajták jellemzı küllemi és termelési tulajdonságai. Magyar Mezőgazdaság. Magyar Juhászat+ Kecsketenyésztés. 2005; 14 (9): 10–11.

[6] Kukovics S. A kecsketenyésztés támogatási lehetıségei, vagy amit szeretnénk. Magyar Mezőgazdaság. Magyar Juhászat+ Kecsketenyésztés. 2006; 15(10): 7–12.

[7] Kukovics S. A kiskérıdzı ágazato idıszerő tenyésztési kérdései. Magyar Mezőgazdaság. Magyar Juhászat + Kecsketenyésztés. 2001; 10(7): 4–8.

[8] Rédecsi Á. A nagyüzemi kecsketenyésztés hazai termelési eredményeinek értékelése két nagyüzemben. 1984; Szakdolgozat, Állatorvos Tudományi Egyetem, Budapest

[9] Hungarian Sheep and Goat Breeding Association. Magyar Juh- és Kecsketenyésztő Szövetség [Internet] 2017. Available from: https://mjksz.hu/magunkrol

[10] Bishop SC. Possibilities to breed for resistance to nematode parasite infections in small ruminants in tropical production systems. Animal. 2012; 6(5): 741–747. doi: 10.1017/S1751731111000681 22558922 10.1017/S1751731111000681

[11] Cremonesi P, Capoferri R, Pisoni G, Del Corvo M, Strozzi F, Rupp R, et al Response of the goat mammary gland to infection with Staphylococcus aureus revealed by gene expression profiling in milk somatic and white blood cells. BMC Genomics. 2012; 13:540 doi: 10.1186/1471-2164-13-540 23046560 10.1186/1471-2164-13-540 PMC3532242

[12] Benavides MV, Sonstegard TS, Kemp S, Mugambi JM, Gibson JP, Baker RL, et al Identification of Novel Loci Associated with Gastrointestinal Parasite Resistance in a Red Maasai x Dorper Backcross Population. PLoS ONE. 2015; 10(4): e0122797 doi: 10.1371/journal.pone.0122797 25867089 10.1371/journal.pone.0122797 PMC4395112

[13] Bergonier D, Berthelot X. New advances in epizootiology and control of ewe mastitis. Livest Prod Sci. 2003; 79(1): 1–16.

[14] Bishop SC, Axford RFE, Nicholas FW, Owen JB. Breeding for disease resistance in farm animals 3rd ed. CABI Publishing; 2010 doi: 10.1079/9781845935559.0000

[15] van Houtert MF, Sykes AR. Implications of nutrition for the ability of ruminants to withstand gastrointestinal nematode infections. Int J Parasitol. 2010; 26(11): 1151–1167. 10.1016/s0020-7519(96)00120-8 9024860

[16] Badaso T, Addis M. Small ruminants haemonchosis: prevalence and associated risk factors in Arsi Negelle municipal abattoir. Ethiopia Glob Vet. 2015; 15(3):315–320. doi: 10.5829/idosi.gv.2015.15.03.96108

[17] FAO/IAEA Agriculture Biotechnology Laboratory–Handbook of Laboratory Exercises. IAEA Laboratories, Seibersdorf, Austria; 2004; pp 18.

[18] Zsolnai A, Orbán L. Accelerated separation of random complex DNA patterns in gels: comparing the performance of discontinuous and continuous buffers. Electrophoresis. 1999; 7: 1462–1468. 10.1002/(SICI)1522-2683(19990601)20:7<1462::AID-ELPS1462>3.0.CO;2-0 10424469

[19] Cappuccio I, Pariset L, Ajmone-Marsan P, Dunner S, Cortes O, Erhardt G, et al Allele frequencies and diversity parameters of 27 single nucleotide polymorphisms within and across goat breeds. Mol Ecol Notes. 2006; 6(4):992–997. doi: 10.1111/j.1471-8286.2006.01425.x

[20] Piras G, Pazzola M, Balia F, Pira E, Dettori ML, Carcangiu V, Vacca GM. Polymorphism of caprine SLC11A1 gene and relationships with hygienic characteristics of milk. Agriculturae Conspectus Scientificus. 2011; 76: 175–178.

[21] Brenaut P, Lefèvre L, Rau A, Laloë D, Pisoni G, Moroni P, et al Contribution of mammary epithelial cells to the immune response during early stages of a bacterial infection to Staphylococcus aureus. Vet Res. 2014; 45:16 doi: 10.1186/1297-9716-45-16 24521038 10.1186/1297-9716-45-16 PMC3937043

[22] Bhuiyan AA, Li J, Wu Z, Ni P, Adetula AA, Wang H, et al Exploring the Genetic Resistance to Gastrointestinal Nematodes Infection in Goat Using RNA-Sequencing. Int J Mol Sci. 2017; 18(4), 751 doi: 10.3390/ijms18040751 28368324 10.3390/ijms18040751 PMC5412336

[23] Raymond M, Rousset F. GENEPOP (version 1.2): population genetics software for exact tests and ecumenicism. J Hered. 1995; 86:248–249. doi: 10.1093/oxfordjournals.jhered.a111573

[24] Yeh FC, Yand RC, Boyle T. POPGENE version 1.32: Microsoft Windows–based freeware for population genetic analysis, quick user guide. Center for International Forestry Research, University of Alberta, Edmonton, Alberta, Canada, 1999

[25] Graves H, Rayburn AL, Gonzalez-Hernandez JL, Nah G, Kim D-S, Lee DK. Validating DNA polymorphisms using KASP assay in Prairie cordgrass (Spartina pectinate Link) populations in the U.S. Front Plant Sci. 2016; 6:1271 doi: 10.3389/fpls.2015.01271 26834772 10.3389/fpls.2015.01271 PMC4722126

[26] Semagn K, Babu R, Hearne S, Olsen M. Single nucleotide polymorphism genotyping using Kompetitive Allele Specific PCR (KASP): overview of the technology and its application in crop improvement. Mol Breed. 2014; 33:1–14. doi: 10.1007/s11032-013-9917-x

[27] Arce I, Martinez-Munoz L, Roda-Navarro P, Fernandez-Ruiz E. The human C-type lectin CLECSF8 is a novel monocyte/macrophage endocytic receptor. Eur J Immunol 2004; 34: 210–220. doi: 10.1002/eji.200324230 14971047 10.1002/eji.200324230

[28] Han B, Mura M, Andrade CF, Okutani D, Lodyga M, dos Santos CC, et al TNFalpha-induced long pentraxin PTX3 expression in human lung epithelial cells via JNK. J Immunol. 2005; 175(12): 8303–8311. doi: 10.4049/jimmunol.175.12.8303 16339571 10.4049/jimmunol.175.12.8303

[29] Salvatori G, Campo S. Current understanding of PTX3 protective activity on Aspergillus fumigatus infection. Med Mycol. 2012; 50(3): 225–233. doi: 10.3109/13693786.2011.648215 22309253 10.3109/13693786.2011.648215

[30] Filipe J., Bronzo V., Curone G., Pollera C., Turin L., Dall’Ara P, et al. PTX3 is up-regulated in epithelial mammary cells during S. aureus intra-mammary infection in goat. Proceeding of Veterinary and Animal Science Days. 2015. 15th- 17th July, Milan, Italy.

[31] Krebs DL, Hilton DJ. SOCS proteins: negative regulators of cytokine signalling. Stem Cells. 2001; 19(5): 378–387. doi: 10.1634/stemcells.19-5-378 11553846 10.1634/stemcells.19-5-378

[32] Raven LA, Cocks BG, Goddard ME, Pryce JE, Hayes BJ. Genetic variants in mammary development, prolactin signalling and involution pathways explain considerable variation in bovine milk production and milk composition. Genet Sel Evol. 2014; 46: 29 doi: 10.1186/1297-9686-46-29 24779965 10.1186/1297-9686-46-29 PMC4036308

[33] Strillacci MG, Frigo E, Schiavini F, Samoré AB, Canavesi F, Vevey M, et al Genome-wide association study for somatic cell score in Valdostana Red Pied cattle breed using pooled DNA. BMC Genetics. 2014; 15:106 doi: 10.1186/s12863-014-0106-7 25288516 10.1186/s12863-014-0106-7 PMC4198737

[34] Wang H, Zhang L, Cao J, Wu M, Ma X, Liu Z, et al Genome-wide specific selection in three domestic sheep breeds. PLoS ONE. 2015; 10:e0128688 doi: 10.1371/journal.pone.0128688 26083354 10.1371/journal.pone.0128688 PMC4471085

[35] Notebaert S, Demon D, Vanden Berghe T, Vandenabeele P, Meyer E. Inflammatory mediators in Escherichia coli-induced mastitis in mice. Comp Immunol Microbiol Infect Dis. 2008; 31: 551–565. doi: 10.1016/j.cimid.2007.10.004 18243314 10.1016/j.cimid.2007.10.004

[36] Hedges JC, Singer CA, Gerthoffer WT. Mitogen-activated protein kinases regulate cytokine gene expression in human airway myocytes. Am J Respir Cell Mol Biol 2000; 23(1): 86–94. doi: 10.1165/ajrcmb.23.1.4014 10873157 10.1165/ajrcmb.23.1.4014

[37] Russo RC, Garcia CC, Teixeira MM, Amaral FA. The CXCL8/IL-8 chemokine family and its receptors in inflammatory diseases. Expert Rev Clin Immunolo. 2014; 10(5): 593–619. doi: 10.1586/1744666X.2014.894886 24678812 10.1586/1744666X.2014.894886

[38] Cotton JA, Platnich JM, Muruve DA, Jijon HB, Buret AG, Beck PL. Interleukin-8 in gastrointestinal inflammation and malignancy: induction and clinical consequences. Int J Interferon Cytokine Mediat Res. 2016; 8: 13–34.

[39] Griesbeck-Zilch B, Osman M, Kuhn C, Schwerin M, Bruckmaier RH, Pfaffl MW, et al Analysis of key molecules of the innate immune system in mammary epithelial cells isolated from marker-assisted and conventionally selected cattle. J Dairy Sci. 2009; 92: 4621–4633. doi: 10.3168/jds.2008-1954 19700725 10.3168/jds.2008-1954

[40] Jiang ZD, Okhuysen PC, Guo DC, He R, King TM, DuPont HL, et al Genetic susceptibility to enteroaggregative Escherichia coli diarrhea: polymorphism in the interleukin-8 promotor region. J Infect Dis. 2003; 188(4): 506–511. doi: 10.1086/377102 12898436 10.1086/377102

[41] Ye BD, Kim SG, Park JH, Kim JS, Jung HC, Song IS. The interleukin- 8–251 A allele is associated with increased risk of noncardia gastric adenocarcinoma in Helicobacter pylori-infected Koreans. J Clin Gastroenterol. 2009; 43(3):233–239. doi: 10.1097/MCG.0b013e3181646701 18542040 10.1097/MCG.0b013e3181646701

[42] Gentile DA, Doyle WJ, Zeevi A, Howe- Adams J, Kapadia S, Trecki J, et al Cytokine gene polymorphisms moderate illness severity in infants with respiratory syncytial virus infection. Hum Immunol. 2003; 64(3): 338–344 12590978 10.1016/s0198-8859(02)00827-3

[43] Tackey E, Lipsky PE, Illei GG. Rationale for interleukin-6 blockade in systemic lupus erythematosus. Lupus. 2004; 13 (5): 339–43, doi: 10.1191/0961203304lu1023oa 15230289 10.1191/0961203304lu1023oa PMC2014821

[44] E GX, Na RS, Zhao YJ, Ma YH, Huang YF. Diversity of TNF-α region in Chinese domestic goats. Genet Mol Res. 2015; 14(2): 3601–3605, doi: 10.4238/2015.April.17.9 25966128 10.4238/2015.April.17.9

[45] Shafer AB, Fan CW, Côté SD Coltman DW. (Lack of) genetic diversity in immune genes predates glacial isolation in the North American mountain goat (Oreamnos americanus). J Hered. 2012; 103: 371–379. doi: 10.1093/jhered/esr138 22268162 10.1093/jhered/esr138

[46] Blum JW, Dosogne H, Hoeben D, Vangroenweghe F, Hammon HM, Bruckmaier RM, et al Tumor necrosis factor-[alpha] and nitrite/nitrate responses during acute mastitis induced by Escherichia coli infection and endotoxin in dairy cows. Domest Anim Endocrinol. 2000; 19: 223–235. 11118787 10.1016/s0739-7240(00)00079-5

[47] Perrier S, Darakhshan F, Hajduch E. IL-1 receptor antagonist in metabolic diseases: Dr Jekyll or Mr Hyde?. FEBS Lett. 2006; 580(27): 6289–94. doi: 10.1016/j.febslet.2006.10.061 17097645 10.1016/j.febslet.2006.10.061

[48] National Center for Biotechnology Information (NCBI) [Internet] 2018 [cited 20 Feb 2018]. Available from: https://www.ncbi.nlm.nih.gov/gene/3557

[49] El-Omar EM, Carrington M, Chow WH, McColl KE, Bream JH, Young HA, et al Interleukin-1 polymorphisms associated with increased risk of gastric cancer. Nature. 2000; 404(6776): 398–402. doi: 10.1038/35006081 10746728 10.1038/35006081

[50] Fumagalli M, Pozzoli U, Cagliani R, Comi RP, Riva S, Clerici M, et al Parasites represent a major selective force for interleukin genes and shape the genetic predisposition to autoimmune conditions. J Exp Med. 2009; 206(6): 1395–1408. doi: 10.1084/jem.20082779 19468064 10.1084/jem.20082779 PMC2715056

[51] Pariset L, Cuteri A, Ligda C, Ajmone-Marsan P, Valentini A, & Econogene Consortium. Geographical patterning of sixteen goat breeds from Italy, Albania and Greece assessed by Single Nucleotide Polymorphisms. BMC Ecol. 2009; 9: 20 doi: 10.1186/1472-6785-9-20 19725964 10.1186/1472-6785-9-20 PMC2754418

[52] Ginja C, Gama LT, Martínez A, Sevane N, Martin-Burriel I, Lanari MR, et al Genetic diversity and patterns of population structure in Creole goats from the Americas. Anim Genet. 2017; 48(3): 315–329. doi: 10.1111/age.12529 28094449 10.1111/age.12529

[53] Kim KS, Yeo JS, Lee JW, Kim JW, Choi CB. Genetic diversity of goats from Korea and China using microsatellite analysis. Asian-Aust J Anim Sci. 2002;15(4): 461–465. doi: 10.5713/ajas.2002.461

