# Supplementary material for: Genetic characterization of indigenous goat breeds in Romania and Hungary with a special focus on genetic resistance to mastitis and gastrointestinal parasitism based on 40 SNPs
Source: PLoS One. 2018 May 9;13(5):e0197051. doi: 10.1371/journal.pone.0197051 (PMC5942826; doi:10.1371/journal.pone.0197051)
Supplement: S1 Table — (DOC) [file pone.0197051.s001.doc]

**Table S1. The collection sites and geographic coordinates of the Romanian and Hungarian goat breeds included in the study.**

| Breed | Number of samples | Collection sites | Geographic coordinates |
| --- | --- | --- | --- |
| Romanian Banat’s White  (n=36) | 12 | Lugoj, Timiş county, RO | 45°41′10″N 21°54′2″E |
|  | 12 | Secaş, Timiş county, RO | 45°54′31″N 21°48′34″E |
|  | 12 | Sânandrei, Timiş county, RO | 45°54′38″N 21°09′51″E |
| Romanian Carpatina  (n=35) | 7 | Secaş, Timiş county, RO | 45°54′31″N 21°48′34″E |
|  | 7 | Arad, Arad county, RO | 46°10′36″N 21°18′4″E |
|  | 7 | Nădlac, Arad county, RO | 46°10′0″N 20°45′2″E |
|  | 7 | Caransebeş, Caraş county, RO | 45°25′17″N 22°13′19″E |
|  | 7 | Sânandrei, Timiş county, RO | 45°54′38″N 21°09′51″E |
| Hungarian Milking  (n=79) | 59 | Ceglédbercel, Pest County, HU | 47°13′21″N 19°39′59″E |
|  | 20 | Káva, Pest County, HU | 47°21′19″N 19°35′16″E |
