# Supplementary material for: Genetic characterization of indigenous goat breeds in Romania and Hungary with a special focus on genetic resistance to mastitis and gastrointestinal parasitism based on 40 SNPs
Source: PLoS One. 2018 May 9;13(5):e0197051. doi: 10.1371/journal.pone.0197051 (PMC5942826; doi:10.1371/journal.pone.0197051)
Supplement: S2 Table — (DOCX) [file pone.0197051.s002.docx]

**Table S2. Details of genes, chromosome location and genomic location at 52 SNP loci under study.**

| **SNP ID** | **Locus** | **Gene Name** | **Chromosome** | **Position** | **Alleles** | **Remarks** |
| --- | --- | --- | --- | --- | --- | --- |
| rs669680484 | PTX3 | pentraxin 3 | 1 | 108076746 | C/T | intron variant |
| rs649383860 | PTX3 | pentraxin 3 | 1 | 108076533 | C/T | intron variant |
| rs648674140 | PTX3 | pentraxin 3 | 1 | 108081598 | C/G | intron variant |
| rs646088457 | PTX3 | pentraxin 3 | 1 | 108076881 | A/G | intron variant |
| rs641385611 | PTX3 | pentraxin 3 | 1 | 108081185 | A/G | intron variant |
| rs654571561 | ICOSLG | inducible T-cell costimulator ligand | 1 | 141881781 | A/G | missense |
| rs651789072 | ICOSLG | inducible T-cell costimulator ligand | 1 | 141882944 | G/T | missense |
| rs651407277 | ICOSLG | inducible T-cell costimulator ligand | 1 | 141881739 | A/G | missense |
| rs644393790 | ICOSLG | inducible T-cell costimulator ligand | 1 | 141880385 | C/T | missense |
| rs666944028 | SLC11A1 | solute carrier family 11 member 1 | 2 | 105766113 | C/T | missense |
| rs652170831 | SLC11A1 | solute carrier family 11 member 2 | 2 | 105764907 | A/G | Intron variant |
| rs643200957 | SLC11A1 | solute carrier family 11 member 3 | 2 | 105763369 | C/T | missense |
| rs646307174 | IL6 | interleukin-6 | 4 | 29257937 | C/T | 3′UTR |
| rs655338449 | CLEC4E | C-type lectin domain family 4 member E | 5 | 93527308 | C/T | missense |
| rs648586705 | CLEC4E | C-type lectin domain family 4 member E | 5 | 93531982 | C/G | missense |
| rs640597911 | CLEC4E | C-type lectin domain family 4 member E | 5 | 93531862 | A/G | missense |
| rs669986850 | CLEC4E | C-type lectin domain family 4 member E | 5 | 93538087 | C/T | upstream variant 2KB |
| rs667413402 | IL8 | interleukin 8 | 6 | 86041868 | A/G | missense |
| rs665173888 | IL8 | interleukin 8 | 6 | 86040123 | A/G | missense |
| rs664817262 | IL4 | interleukin 4 | 7 | 18135746 | A/G | missense |
| rs669391945 | TLR4 | toll-like receptor 4 | 8 | 105007687 | A/C | missense |
| rs665886793 | IL1RN | interleukin 1 receptor antagonist | 11 | 46349648 | A/G | missense |
| rs659842900 | IL1RN | interleukin 1 receptor antagonist | 11 | 46358256 | A/G | missense |
| rs640582069 | IL1RN | interleukin 1 receptor antagonist | 11 | 46353777 | G/T | missense |
| rs638939037 | IL1RN | interleukin 1 receptor antagonist | 11 | 46358191 | C/T | missense |
| rs653303066 | IL1B | interleukin 1 beta | 11 | 46048631 | C/T | missense |
| rs640194180 | IL1B | interleukin 1 beta | 11 | 46047709 | A/G | missense |
| rs661943224 | IL15RA | interleukin 15 receptor subunit alpha | 13 | 10343904 | C/T | missense |
| rs660381568 | IL15RA | interleukin 15 receptor subunit alpha | 13 | 10380224 | C/T | missense |
| rs659726350 | IL15RA | interleukin 15 receptor subunit alpha | 13 | 10343919 | G/T | missense |
| rs655382175 | IL15RA | interleukin 15 receptor subunit alpha | 13 | 10354737 | A/G | missense |
| rs653428028 | IL15RA | interleukin 15 receptor subunit alpha | 13 | 10346298 | A/G | missense |
| rs648293427 | IL15RA | interleukin 15 receptor subunit alpha | 13 | 10344025 | C/T | missense |
| rs647408958 | IL15RA | interleukin 15 receptor subunit alpha | 13 | 10354726 | A/G | missense |
| rs639466960 | IL15RA | interleukin 15 receptor subunit alpha | 13 | 10380214 | C/T | missense |
| rs638858680 | IL15RA | interleukin 15 receptor subunit alpha | 13 | 10380115 | A/G | missense |
| rs635969404 | IL15RA | interleukin 15 receptor subunit alpha | 13 | 10354813 | C/T | missense |
| rs637266882 | IL15 | interleukin 15 | 17 | 15894920 | C/T | upstream variant |
| rs670223534 | IL15 | interleukin 15 | 17 | 15812147 | A/T | missense, upstream variant |
| rs664582176 | TGFB1 | transforming growth factor beta 1 | 18 | 49357570 | A/G | missense |
| rs639895207 | TGFB1 | transforming growth factor beta 1 | 18 | 49347870 | C/T | missense |
| rs669561078 | TNFSF13 | TNF superfamily member 13 | 19 | 26523480 | A/G | missense |
| rs669547621 | MAP3K14 | mitogen-activated protein kinase kinase kinase 14 | 19 | 44066584 | C/T | missense |
| rs668924310 | MAP3K14 | mitogen-activated protein kinase kinase kinase 14 | 19 | 44050352 | C/T | missense |
| rs661303008 | MAP3K14 | mitogen-activated protein kinase kinase kinase 14 | 19 | 44050265 | A/G | missense |
| rs651811571 | MAP3K14 | mitogen-activated protein kinase kinase kinase 14 | 19 | 44062724 | C/G | missense |
| rs644683905 | MAP3K14 | mitogen-activated protein kinase kinase kinase 14 | 19 | 44050368 | G/T | missense |
| rs637967566 | MAP3K14 | mitogen-activated protein kinase kinase kinase 14 | 19 | 44050379 | C/T | missense |
| rs635583012 | SOCS3 | suppressor of cytokine signaling 3 | 19 | 52626440 | G/T | intron variant |
| rs661165283 | TNF | tumor necrosis factor | 23 | 26141981 | A/T | missense |
| rs650731617 | IL4R | interleukin-4 receptor | 25 | 24974944 | C/T | missense |
| rs661914424 | TLR3 | toll-like receptor 3 | 27 | 14987931 | C/G | missense |
