# Supplementary material for: Genetic characterization of indigenous goat breeds in Romania and Hungary with a special focus on genetic resistance to mastitis and gastrointestinal parasitism based on 40 SNPs
Source: PLoS One. 2018 May 9;13(5):e0197051. doi: 10.1371/journal.pone.0197051 (PMC5942826; doi:10.1371/journal.pone.0197051)
Supplement: S3 Table — (DOCX) [file pone.0197051.s003.docx]

**Table S3. Success ratio of 52 SNPs investigated through KASP assay for 150 samples from Banat’s White, Carpatina and Hungarian Milking goat breeds**

| Status | Number | Success ratio | SNP / Gene |
| --- | --- | --- | --- |
| Polymorphic | 16 | 30.77% | rs669680484 / *PTX3*, rs646307174 / *IL6*, rs655338449 / *CLEC4E*, rs669986850 / *CLEC4E*, rs667413402 / *IL8*, rs665173888 / *IL8*, rs659842900 / *IL1RN*, rs640582069 / *IL1RN*, rs661943224 / *IL15RA*, rs648293427 / *IL15RA*, rs647408958 / *IL15RA*, rs635969404 / *IL15RA*, rs669561078 / *TNFSF13*, rs635583012 / *SOCS3*, rs661165283 / *TNF*, rs661914424 / *TLR3* |
| Monomorphic | 24 | 46.15% | rs649383860 / *PTX3,* rs648674140 / *PTX3,* rs646088457 / *PTX3,* rs641385611 / *PTX3,* rs651407277 / *ICOSLG,* rs644393790 / *ICOSLG,* rs666944028 / *SLC11A1,* rs643200957 / *SLC11A1,* rs648586705 / *CLEC4E,* rs640597911 / *CLEC4E,* rs669391945 / *TLR4,* rs665886793 / *IL1RN*, rs653303066 / *IL1B,* rs640194180 / *IL1B,* rs660381568 / *IL15RA,* rs655382175 / *IL15RA,* rs639466960 / *IL15RA,* rs638858680 / *IL15RA,* rs637266882 / *IL15,* rs664582176 / *TGFB1,* rs639895207 / *TGFB1,* rs668924310 / *MAP3K14,* rs651811571 / *MAP3K14,* rs650731617 / *IL4R* |
| Failed | 12 | 23.08% | rs654571561 / *ICOSLG,* rs651789072 / *ICOSLG,* rs652170831 / *SLC11A1,* rs664817262 / *IL4,* rs638939037 / *IL1RN*, rs659726350 / *IL15RA,* rs653428028 / *IL15RA,* rs670223534 / IL15, rs669547621 / *MAP3K14,* rs661303008 / *MAP3K14,* rs644683905 / *MAP3K14,* rs637967566 / *MAP3K14* |
